# Aberrant expression of PROS1 correlates with human papillary thyroid cancer progression

**DOI:** 10.7717/peerj.11813

**Published:** 2021-08-03

**Authors:** Jing Wang, Minxiang Lei, Zhijie Xu

**Affiliations:** 1 Department of Endocrinology, Xiangya Hospital of Central South University, Changsha, Hunan, China; 2 Department of Pathology, Xiangya Hospital of Central South University, Changsha, Hunan, China; 3 National Clinical Research Center for Geriatric Disorders, Xiangya Hospital of Central South University, Changsha, Hunan, China

**Keywords:** *PROS1*, Papillary thyroid carcinoma, Cell proliferation, Cell migration, Immune-related signaling

## Abstract

**Background:**

Papillary thyroid carcinoma (PTC) is the most common type of thyroid cancer (TC). Considering the important association between cellular immunity and PTC progression, it is worth exploring the biological significance of immune-related signaling in PTC.

**Methods:**

Several bioinformatics tools, such as R software, WEB-based Gene SeT AnaLysis Toolkit (WebGestalt), Database for Annotation, Visualization and Integrated Discovery (DAVID), Search Tool for the Retrieval of Interacting Genes (STRING) and Cytoscape were used to identify the immune-related hub genes in PTC. Furthermore, *in vitro* experiments were adopted to identify the proliferation and migration ability of *PROS1* knockdown groups and control groups in PTC cells.

**Results:**

The differentially expressed genes (DEGs) of five datasets from Gene Expression Omnibus (GEO) contained 154 upregulated genes and 193 downregulated genes, with Protein S (*PROS1*) being the only immune-related hub gene. Quantitative real-time polymerase chain reaction (RT-qPCR) and immunohistochemistry (IHC) have been conducted to prove the high expression of *PROS1* in PTC. Moreover, *PROS1* expression was significantly correlated with lymph nodes classification. Furthermore, knockdown of *PROS1* by shRNAs inhibited the cell proliferation and cell migration in PTC cells.

**Conclusions:**

The findings unveiled the clinical relevance and significance of *PROS1* in PTC and provided potential immune-related biomarkers for PTC development and prognosis.

## Introduction

Thyroid cancer (TC) is the most common endocrine malignancy consisting of three types: differentiated (papillary and follicular TC), undifferentiated (poorly differentiated and anaplastic TC), and medullary TC (Du et al., 2020; Wu et al., 2020; Yu et al., 2020), with the papillary thyroid carcinoma (PTC) being the most common type (Abooshahab et al., 2020; Lou et al., 2020; Meng et al., 2020). Because of the increased sensitivity of diagnostic procedures, PTC, especially microcarcinoma, has been frequently detected over the last decades (Yang et al., 2020; Zhou et al., 2020). Therefore, identifying risk factors for the progression is of great significance for PTC diagnosis and treatment.

Though the complex mechanism leading to PTC remains unclear, the critical role of genetic alteration in PTC biology has been proved in recent years, such as B-type *RAF* kinase (*BRAF*^*V600E*^), telomerase reverse transcriptase (*TERT*), tumor protein P53 (*TP53*), phosphatidylinositol-4,5-bisphosphate 3-kinase catalytic subunit alpha (*PIK3CA*) (Dong et al., 2020; Prete et al., 2020; Ricarte-Filho et al., 2009; Vitale et al., 2017; Xing et al., 2014), and so on. *BRAF*^*V600E*^ represents the most common mutation in PTC (Kim et al., 2010). In addition, emerging studies demonstrated the critical role of cellular immunity in PTC. The relation between thyroid-stimulating hormone (TSH), anti-thyroglobulin antibodies (TgAb) (Boi et al., 2013; Medenica et al., 2015; Xiao et al., 2019), thyroid autoimmunity and the occurrence and development of PTC (Ferrari et al., 2020) has been demonstrated. Several bioinformatics reports have identified the important roles of immune-related biomarkers in PTC pathogenesis, such as AHNAK nucleoprotein 2 (AHNAK2), angiotensin II receptor type 1 (AGTR1), etc. (Li et al., 2021; Lin et al., 2019; Xie et al., 2020b; Xue, Li & Lu, 2020). In patients with PTC, M2 macrophages, Tregs, monocytes, neutrophils, DCs, MCs, and M0 macrophages appeared to play a tumor-promoting role, while M1 macrophages, CD8^+^ T cells, B cells, NK cells, and T follicular helper (TFH) cells might play an anti-tumor part (Xie et al., 2020a). Furthermore, Di Marco et al. (2020) found the potential of *COPI* coat complex subunit zeta 1 (*COPZ1*) for TC treatment because it could inhibit anti-tumor immune response. Besides, the inhibitors of cytotoxic T lymphocyte antigen 4 (*CTLA-4*) and programmed cell death protein-1 (*PD-1*) have been used in clinical trials in TC (Varricchi et al., 2019). Moreover, Liotti et al. (2021) found that *PD-1* could restore anti-cancer immunity and directly impair TC cell growth by inhibiting the mitogen-activated protein kinase (*MAPK*) signaling pathway. However, more studies are required to understand the importance of immune-related mechanisms in PTC.

In this study, the immune-related gene *PROS1* in PTC was identified with comprehensive bioinformatics methods, whose expression was higher in PTC tissues than normal adjacent tissues and was related to the lymph nodes classification. Knockdown of *PROS1* inhibited the proliferation and migration in PTC cells. The findings suggested that *PROS1* could promote PTC progression and may provide a potential biomarker for PTC diagnosis and treatment.

## Materials & Methods

### Microarray data of PTC

A total of five microarray datasets of PTC (GSE3467 (He et al., 2005), GSE3678, GSE29265, GSE33630 (Dom et al., 2012; Tomás et al., 2012) and GSE60542 (Tarabichi et al., 2015)) were downloaded from GEO (Affymetrix GPL570 platform, Affymetrix Human Genome U133 Plus 2.0 Array, Santa Clara, CA, USA, https://www.ncbi.nlm.nih.gov/geo/). GSE3467 consists of nine PTC and nine normal thyroid samples. GSE3678 includes seven PTC and seven normal thyroid samples. GSE29265 is composed of 20 PTC and 20 normal control samples. GSE33630 contains 49 PTC and 45 normal control samples. GSE60542 is comprised of 33 PTC and 30 normal control samples.

### Identification of DEGs

The language software R (version 3.5.1, https://www.r-project.org/) was used in DEGs screening. Background correction for the CEL microarray data was performed, expression of quartile data was normalized and calculated with robust multi-array average (RMA) method in *affy* package (http://www.bioconductor.org/packages/release/bioc/html/affy.html) (Gautier et al., 2004), and the DEGs between PTC and normal control samples were identified using the *limma* package (http://www.bioconductor.org/packages/release/bioc/html/limma.html) (Ritchie et al., 2015) in R. The normalization graphs of the five datasets were shown in Fig. S1. Additionally, *P*-value < 0.05 and |log2 (fold change) > 1 were considered as the threshold for identifying DEGs (Xu et al., 2021; Yan et al., 2019b; Zhang et al., 2019). For visualization, the Venn diagrams of the five datasets were drawn with the online tool: Venn diagrams (http://bioinformatics.psb.ugent.be/webtools/Venn/), and volcano plots of DEGs were drawn by *ggplot2* package in R.

**Figure 1:**
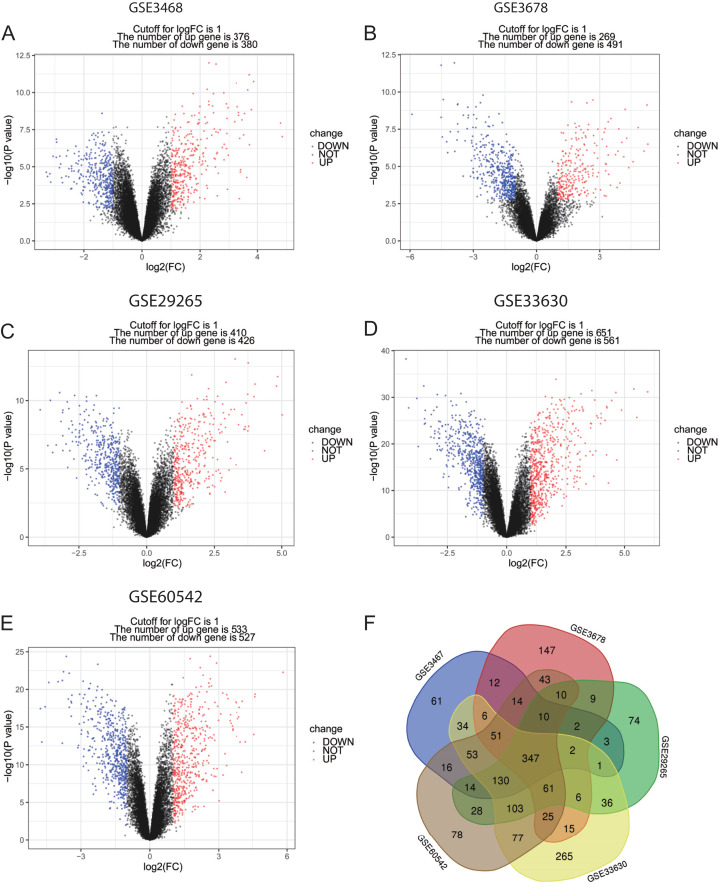
Volcano plots and Venn diagram of DEGs. (A)–(E) Volcano plot of GSE3467, GSE3678, GSE29265, GSE33630 and GSE60542. Red points indicate upregulated genes, blue points indicate downregulated genes, and black points indicate genes with unchanged expression. (F) Venn diagram of DEGs of the five datasets: DEGs were selected with criteria: |fold change| > 1 and *P*-value < 0.05 in the five datasets, and the intersection of DEGs of the five datasets consisted of 347 DEGs.

### Enrichment analysis of DEGs

Gene ontology (GO) consists of cellular component (CC), biological process (BP), and molecular function (MF). In this study, GO enrichment analysis was performed using the WEB-based Gene SeT AnaLysis Toolkit (WebGestalt) (http://www.webgestalt.org/) (Wang et al., 2017) with FDR = 0.05 being the threshold of statistical significance. Besides, the Kyoto Encyclopedia of Genes and Genomes (KEGG) pathway analysis was conducted by Visualization and Integrated Discovery (DAVID, version 6.8) (http://david.ncifcrf.gov) (Huang et al., 2007), with a threshold of *P* < 0.05 and false discovery rate (FDR) < 0.25. WebGestalt and ImageGP (http://www.ehbio.com/ImageGP/) were adopted to visualize GO and KEGG enrichment analysis.

### PPI network construction and modules selection

Search Tool for the Retrieval of Interacting Genes (STRING) (http://string-db.org) (version 10.0) (Szklarczyk et al., 2015) was used to predict the protein–protein interaction (PPI) network of DEGs. Cytoscape (version 3.6.1) (Demchak et al., 2014) was applied to plot the PPI network and the significant modules of the PPI network were confirmed with Molecular Complex Detection (MCODE), a plug-in for Cytoscape. The selection criteria were as below: degree cut-off = 2, node score cut-off = 0.2, Max depth = 100 and k-score = 2.

### Hub genes and immune-related gene selection

Hub genes were selected from the significant modules with the criterion of MCODE Score ≥ 5, and the immune-related genes of hub genes were further confirmed using InnateDB (https://www.innatedb.com/), a publicly available database for identifying the genes, proteins, and signaling pathways involved in the innate immune response (Breuer et al., 2013).

### RNA extraction and quantitative real-time polymerase chain reaction (RT-qPCR)

A total of 13 pairs of PTC and normal adjacent tissues were obtained from PTC patients who had surgical operations from June 2020 to August 2020 in Xiangya Hospital of Central South University, China. Papillary thyroid carcinoma was diagnosed by two pathologists from the Pathology Department of Xiangya Hospital. Ethical approval of this study was obtained from the Ethics Committee of Xiangya Hospital of Central South University (Number: 202005059) and the informed consent form (IFC) was collected from each patient involved. The PTC and paired normal adjacent tissues were immediately stored in liquid nitrogen immediately after surgery for the total RNA extraction with TRIzol reagent following the Invitrogen manufacturer’s protocol. The Nanodrop ND-8000 Spectrophotometer (Thermo Fisher Scientific Waltham, MA, USA) was used to detect the quality and quantity of total RNA. The criterion for the extracted pure RNA was an A260/A280 of 1.8 to 2.1. The isolated RNA concentration of all the samples was normalized with RNase-free water and then mRNA was reverse-transcribed into cDNA by All-in-One™ First-Strand cDNA Synthesis Kit (GeneCopoeia, Rockville, MD, USA). The cDNA samples were stored at −20 °C before use. Then RT-qPCR was conducted with All-in-One™ qPCR Mix (GeneCopoeia, Rockville, MD, USA) and specific primers. The procedure was as follows: firstly, denaturation at 95 °C for 10 min, then 40 cycles of denaturation at 95 °C for 10 s, followed by annealing at 62.5 °C for 20 s and extension at 72 °C for 32 s. *β-actin* served as the control and the fold change of the *PROS1* expression in PTC and normal adjacent samples was calculated with 2^−ΔΔCt^ method (Buford et al., 2020; Pandelides et al., 2020). The sequences of the primers of *PROS1* and *β-actin* were synthesized by Sangon Biotech (Shanghai, China), as listed in Table 1.

**Table 1:** Sequences of primers for RT-qPCR.

Gene symbol	Primer sequence
*PROS1*	F: 5′-CCATTCCAGACCAGTGTAG−3′
	R: 5′-GGTAACTTCCAGGTGTATTATC−3′
*β-actin*	F: 5′-CCTGGCACCCAGCACAAT−3′
	R: 5′-GGGCCGGACTCGTCATAC−3′

### Immunohistochemistry (IHC)

A total of 118 PTC and 92 normal adjacent tissues paraffin specimens were obtained from Xiangya Hospital of Central South University. The ethical approval of this study was gained from the Ethics Committee of Xiangya Hospital of Central South University (Number: 202005059). IHC was conducted with reference to the manufacturer’s protocol: 1. Deparaffinization and rehydration; 2. Antigen retrieval: the de-paraffinized sections were placed in 10 mM Sodium Citrate buffer (pH 6.0) and then were held at sub-boiling temperature for 10 min; 3. Blocking endogenous target activity and nonspecific sites; 4. Antibody staining with a rabbit antibody of *PROS1* (1:500; Novus Biologicals, Littleton, CO, USA) that was then incubated at 37 °C for 2 h. After washing with PBS for 10 min, slides were added with a secondary antibody for 30 min (ZSGB-Bio Origene, Beijing, China); 5. Counterstain with hematoxylin and dehydration. All the stained sections of the slides were observed with the light microscope by the pathologist to evaluate the intensity of IHC. The staining intensity of the protein expression was scored following the criteria (Yan et al., 2019b) as follows: 0 (negative), 1 (weak expression), 2 (moderate expression), and 3 (strong expression). The percentage of cytoplasmic expression cells was scored as the following: 0 (percentage ≤ 10), 1 (percentage 11–25), 2 (percentage 26–50), 3 (percentage 51–75) and 4 (percentage > 75). The final score was the product of intensity score and a percentage score, and scores ranging from 0 to 7 were classified as low expression while scores ranging 8 to 12 were classified as high expression.

### Western blot

Cells were washed twice with PBS, and whole cell lysed with ice‑cold RIPA lysis buffer (Beyotime Institute of Biotechnology, Jiangsu, China), protease Inhibitor (1:100, Biomake, Cambridge, MA, USA) were added freshly. Protein was determined using the BCA method (Thermo Fisher, Waltham, MA, USA) following the manufacturer’s instructions. Equal protein amounts were separated by SDS-PAGE on 8% Tris-glycine, and then transferred to PVDF membranes (Millipore, Burlington, MA, USA) blocked with 5% skim milk for 2 h at room temperature, and incubated with primary antibody *PROS1* (1:1,000; Novus Biologicals, USA) and α-tubulin (1:1,000; ProteinTech, Rosemont, IL, USA) overnight at 4 °C. The next day the membrane was washed three times in 1 × TBST for 10 min and incubated with horseradish peroxidase (HRP)‑conjugated AffiniPure goat anti‑rabbit (dilution 1:5,000; ProteinTech, Rosemont, IL, USA) for 1 h. After incubation, the membranes were washed three times with 1 × TBS-T. Protein bands were visualized with an enhanced chemiluminescence substrate (Nanjing Vazyme Biological Technology, Nanjing, China). Images were captured using the GeneGenius Image System (Syngene, Frederick, MD, USA).

### Cell culture and cell transfection

Cells were grown in RPMI Medium 1,640 (Gibco, Waltham, MA, USA) supplemented with 10% FBS (Biological Industries, Beit HaEmek, Israel), 1% penicillin and streptomycin (Gibco, Waltham, MA, USA), 1% L-Glutamine, and 1% Sodium Pyruvate 100 mM Solution (Gibco, Waltham, MA, USA), at 37 °C with 5% CO2. BCPAP and KTC-1 cell lines were provided by the Chinese Academy of Sciences Stem Cell Bank. BCPAP and KTC-1 cell lines were infected by lentivirus with *PROS1* knockdown shRNA (shPS1 and shPS2) and Control shRNA (shCON), which Shanghai Genechem of China formulated. The sequences of sh*PROS1* and shCON were provided in Table S1. Stably infected cells were selected by puromycin 2 μg/ml for BCPAP and KTC-1 cell lines.

### Cell proliferation assay

Proliferation assay was performed with the 5-ethynyl-2′-deoxyuridine (EdU) method (Beyotime Institute of Biotechnology, Jiangsu, China). Cells were seeded into 24-well plates (50,000 cells/well) and further cultured at 37 °C for 24 h, which were then washed with PBS, followed by the addition of fresh medium containing 10 µM EdU. Cells were subsequently incubated for 2 h at 37 °C and washed with PBS to remove the EdU and medium. Then the cells were fixed in 4% paraformaldehyde at room temperature for 15 min and washed with PBS again. After incubated with Click Solution for 30 min and then with Hoechst 33,342 (1,000×) for 10 min at room temperature protected from light, positive cells were observed under a fluorescent microscope (Leica, Germany; magnification, ×100). The positive cells were observed with software ImageJ (version 1.43; National Institutes of Health, Bethesda, MD, USA). The numbers of EdU-positive and Hoechst 33,342-positive cells were calculated from three images of each group.

### Cell migration assay

Migration assay was completed by wound-healing assay. Indicated cells were seeded in 6 well plates until the formation of a confluent monolayer, and a “wound” in each well was created by scratching the monolayer with a pipette tip. Cells were washed with PBS and then RPMI Medium 1,640 growth medium was added. The wound was photographed (×100 magnification) at 0 and 24 h, the area of which was then calculated with ImageJ. All wound-healing experiments were performed in triplicate.

### Statistical analysis

The results of RT-qPCR, IHC, cell proliferation assay, and cell migration assay were analyzed with the application of SPSS version 22.0 (IBM Corp, Chicago, IL, USA). GraphPad Prism V.7.0 (GraphPad Software, La Jolla, CA, USA) was applied to draw figures. The results of RT-qPCR were analyzed through paired-samples t-test; the results of cell proliferation and migration assay were analyzed through t-test, and the results of IHC and the association between *PROS1* expression and clinicopathological characteristics of PTC patients were analyzed through the nonparametric test. *P*-value < 0.05 was set as the criterion of statistical significance.

## Results

### Identification of DEGs in PTC

In this study, PTC and normal control samples in GSE3467, GSE3678, GSE29265, GSE33630, and GSE60542 were analyzed. Based on the threshold mentioned above, DEGs (756 in GSE3467, 760 in GSE3678, 836 in GSE29265, 1,212 in GSE33630, and 1,060 in GSE60542) were identified. The volcano plots revealed DEGs of 5 datasets with upregulated genes marked in red and downregulated genes marked in blue (Figs. 1A–1E). A total of 347 consistently dysregulated genes were illustrated in a Venn diagram, including 154 upregulated DEGs and 193 downregulated DEGs (Fig. 1F).

### Enrichment analysis for DEGs

**Figure 2:**
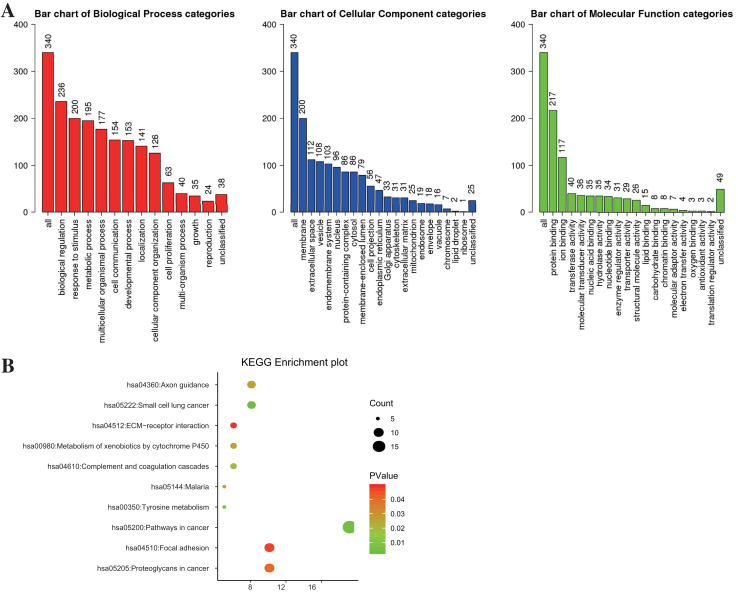
The enrichment analysis for DEGs in PTC. (A) GO enrichment was analyzed by WebGestalt with a threshold of FDR = 0.05. (B) KEGG enrichment was analyzed by DAVID with a threshold of *P* < 0.05 and FDR < 0.25.

WebGestalt and DAVID were adopted to analyze the biological classification of DEGs, the obtained GO (including BP, CC, and MF) and KEGG pathway enrichment analysis results were plotted. As shown in Fig. 2A, DEGs were enriched in BP, including biological regulation, response to the stimulus, and metabolic process. As for CC, DEGs showed an obvious enrichment tendency in the membrane and extracellular space, and for MF, DEGs were mainly enriched in protein binding and ion binding. Furthermore, KEGG pathway analysis indicated that the enrichment was mainly related to the pathway in cancer, proteoglycans in cancer and focal adhesion pathways (Fig. 2B).

### Construction of PPI network and identification of immune-related gene

**Figure 3:**
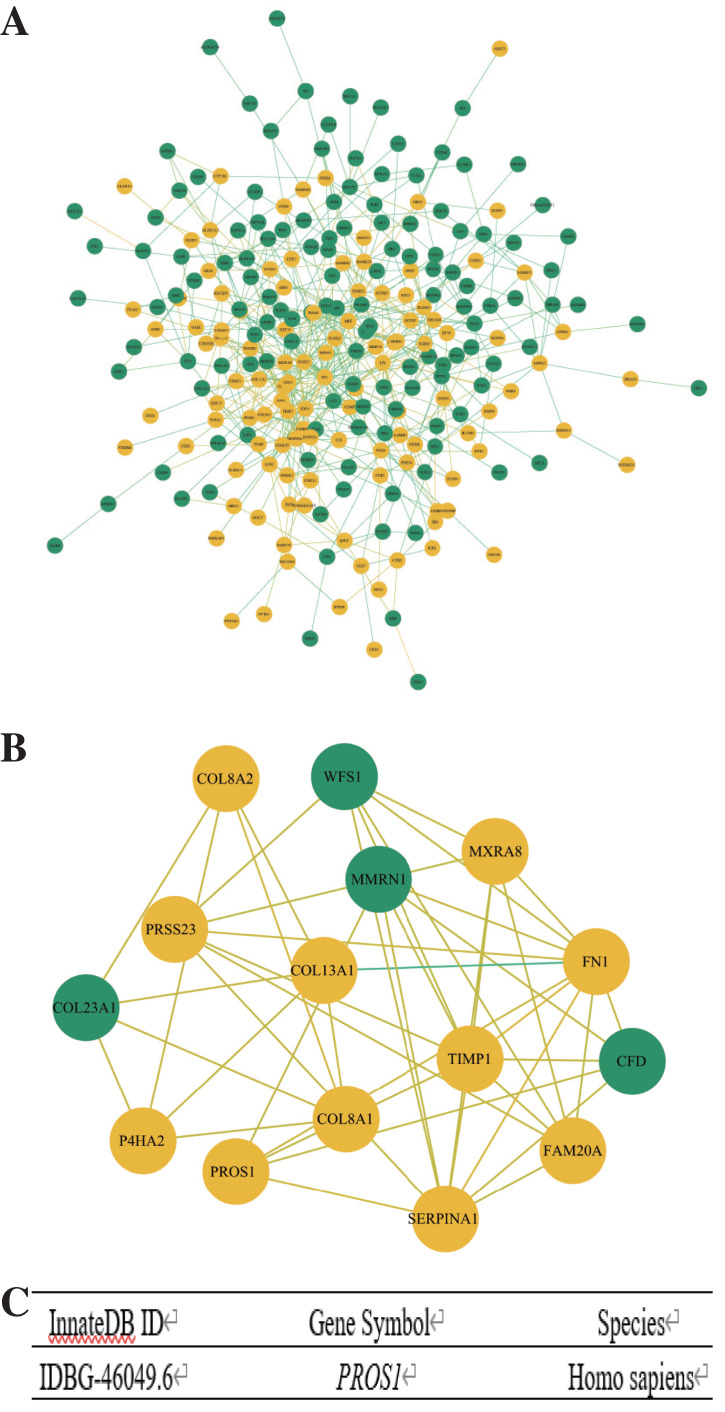
Construction of PPI network and identification of immune-related hub gene. (A) The PPI network of DEGs was completed by Cytoscape: upregulated genes (yellow) and downregulated genes (green). (B) The hub genes were selected from the PPI network utilizing MCODE with the criterion of MCODE Score ≥ 5. Upregulated genes were marked in yellow and downregulated genes were marked in green. (C) InnateDB datebase identified *PROS1* as the candidate innate immune response-associated gene.

The PPI network of DEGs was constructed by Cytoscape (Fig. 3A) and the significant modules were selected for MCODE analysis. Based on the criterion of MCODE Score ≥ 5, 15 hub genes, including 11 upregulated genes and four downregulated genes were selected (Fig. 3B). Subsequently, the gene *PROS1* was identified as the candidate innate immune response-associated gene (Fig. 3C) with innateDB datebase.

### *PROS1* expression analyzed by RT-qPCR

**Figure 4:**
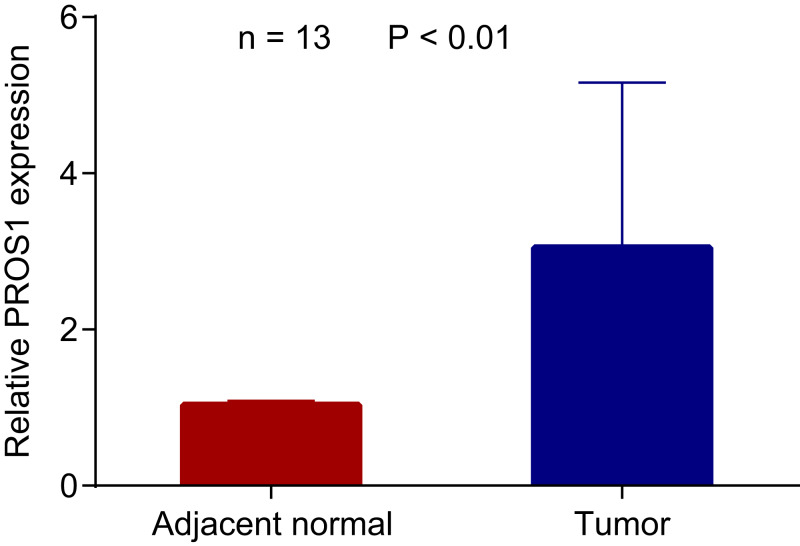
*PROS1* expression in PTC and normal group using RT-qPCR. *PROS1* expression in PTC and normal group using RT-qPCR, *P*-value < 0.01.

The RNA expression of *PROS1* in 13 pairs of PTC and normal adjacent tissues was detected by RT-qPCR. And as shown in Fig. 4, *PROS1* was upregulated in PTC tissues. Similarly, the evaluation of *PROS1* expression in PTC samples from TCGA further illustrated that the expression of *PROS1* was significantly overexpressed in PTC (Fig. S2).

### The expression results of *PROS1* analyzed by IHC

**Figure 5:**
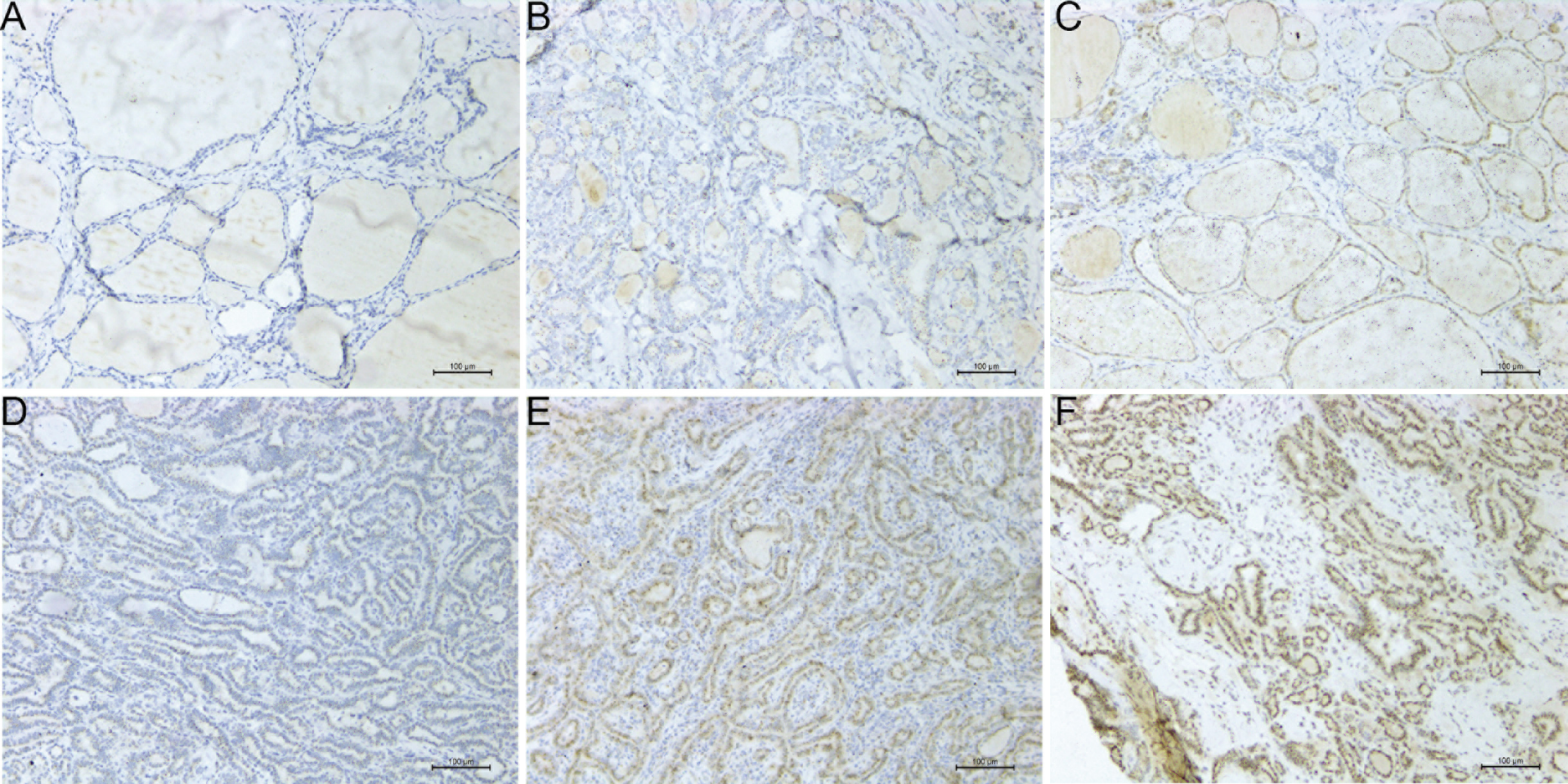
Microscope images showing PROS1 expression of IHC in PTC and normal control tissues (magnification: 200×). Negative (A), weak (B) and moderate intensity (C) of PROS1 expression in the control group. Weak (D), moderate (E) and strong intensity (F) of PROS1 expression in the PTC group.

The IHC expression of PROS1 in 118 PTC and 92 normal adjacent tissues was conducted to compare its protein level in PTC with that in normal tissues. The evaluation standard was described in the materials and methods section. The statistical analysis data showed that PROS1 expression in the PTC group was higher than that in the control group. The results of IHC were summarized in Table 2 and several representative IHC pictures were shown in Figs. 5A–5F.

**Table 2:** Statistical analysis of immunohistochemistry results.

	Control	PTC
Total	92	118
Intensity of negative	49 (53.3%)	14 (11.9%)
Intensity of weak	36 (39.1%)	61 (51.7%)
Intensity of moderate	7 (7.6%)	37 (31.3%)
Intensity of strong	0	6 (5.1%)
Low expression of *PROS1*	86 (93.5%)	76 (64.4%)
High expression of *PROS1*	6 (6.5%)	42 (35.6%)
*P*-value		<0.001

### Relationship between *PROS1* expression and clinicopathological features of PTC patients

The clinicopathological information of patients with PTC has been provided as Table S2. The association between *PROS1* expression and the clinicopathological characteristics of PTC patients was summarized in Table 3. *PROS1* expression was significantly correlated to lymph nodes classification (*P* = 0.02) but not statistically correlated with patients’ age, gender, tumor classification, extrathyroidal invasion, and *BRAF*^*V600E*^ mutation.

**Table 3:** The summary of the relationship between *PROS1* expression and clinicopathological features of PTC patients.

Category	No. patients (%)	*PROS1*		*P*-value
		Low expression	High expression	
Age				0.57
<55	108 (96.4)	69	39	
≥55	4 (3.6)	2	2	
Gender				0.19
Male	33 (29.5)	24	9	
Female	79 (70.5)	47	32	
Stage				0.69
I	110 (98.2)	70	40	
II	2 (1.8)	1	1	
T classification				0.48
pT1	104 (92.8)	65	39	
pT2–T3	8 (7.2)	6	2	
N classification				0.02^#^
pN0	60 (53.6)	32	28	
pN1	52 (46.4)	39	13	
Extrathyroidal invasion				0.83
No	86 (76.8)	55	31	
Yes	26 (23.2)	16	10	
*BRAF*^*V600E*^ Mutation				0.26
No	10 (8.9)	8	2	
Yes	102 (91.1)	63	39	

**Notes:**

The Stage, T classification and N classification refer to the 8th edition of AJCC.

# *P* < 0.05.

### The proliferation and migration of PTC cells suppressed by *PROS1* knockdown

**Figure 6:**
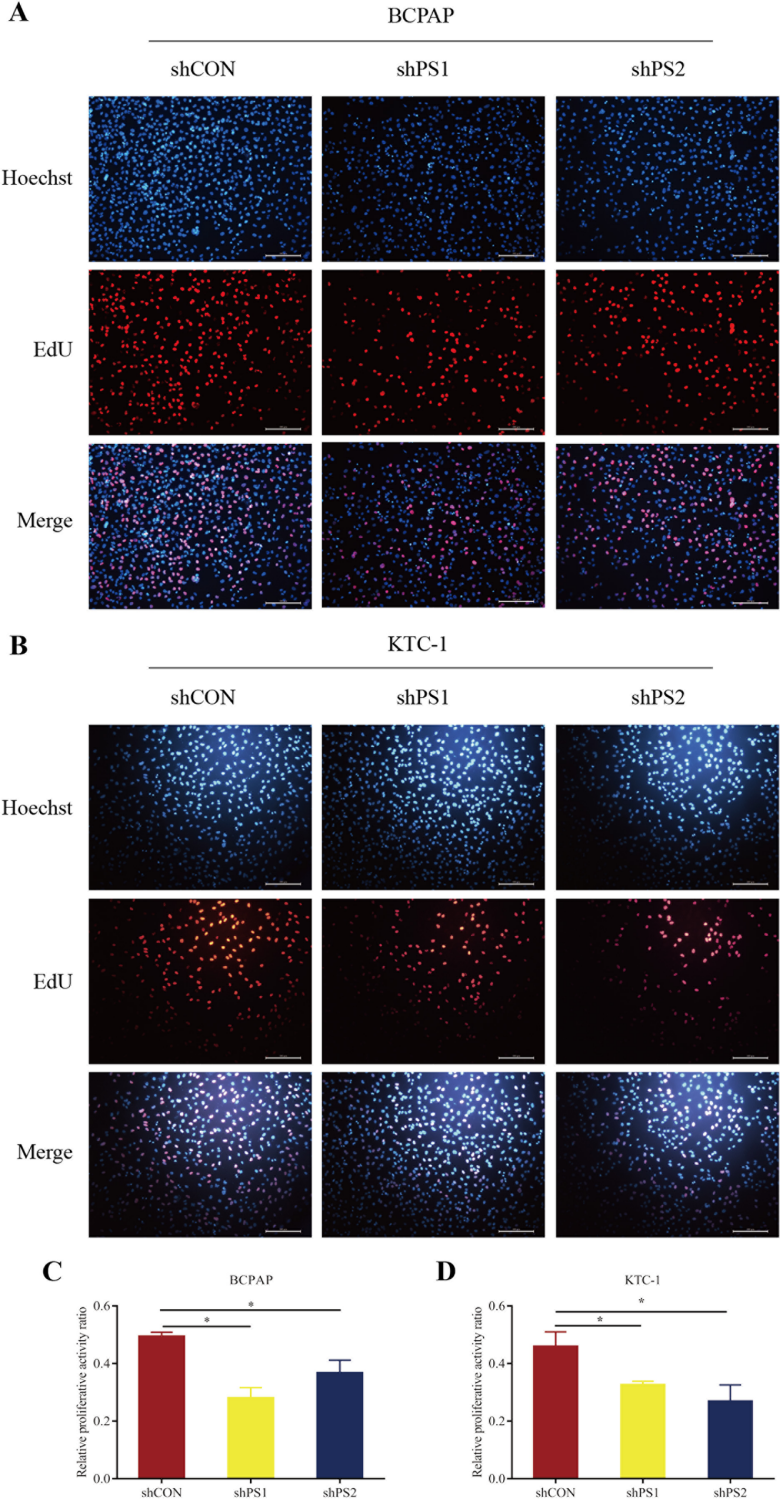
EdU assay of PROS1 knockdown groups and control groups of the two cell lines. (A–B) EdU assay of BCPAP and KTC-1 cells. (C–D) Statistical analysis graphs of EdU assay in BCPAP and KTC-1 cells, *P < 0.05.

**Figure 7:**
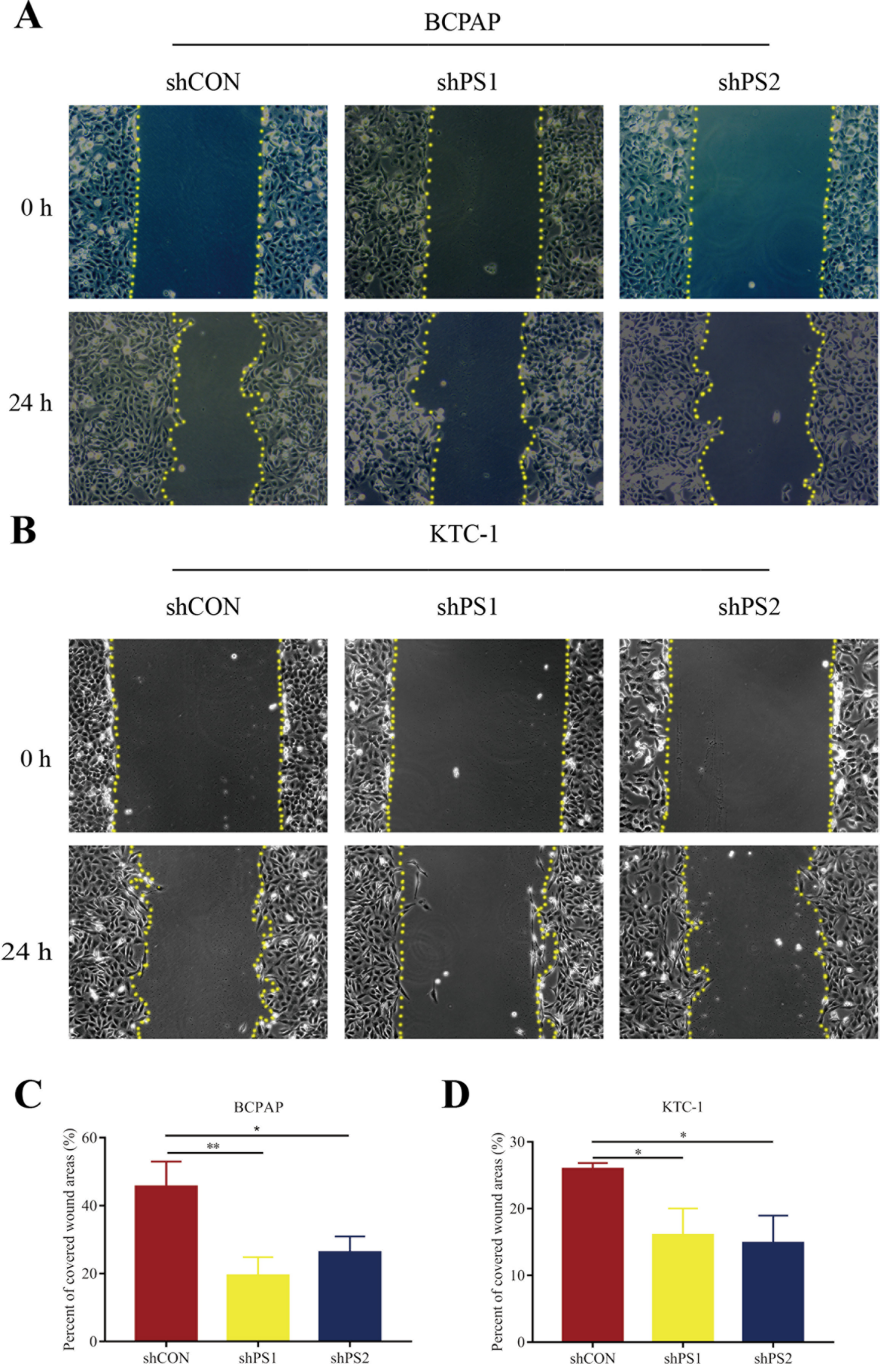
Wound-healing assay of PROS1 knockdown groups and control groups of the two cell lines. (A–B) Wound-healing assay of BCPAP and KTC-1 cells. (C–D) Statistical analysis graphs of the wound-healing assay in BCPAP and KTC-1 cells, *P < 0.05, **P < 0.01.

*PROS1* was knockdown by shRNAs in BCPAP and KTC-1 cell lines. The downregulated expression levels of *PROS1* after treatment with sh*PROS1* (shPS1 and shPS2) were shown in Fig. S3. The results of the EdU assay demonstrated that decreased number of EdU‑positive cells was observed in BCPAP and KTC-1 cells transfected with *PROS1* shRNAs (Figs. 6A–6D). Furthermore, the results of wound-healing assays illustrated a decrease in the percentage of covered scratch area in sh*PROS1*-treated groups compared with the shCON groups in these two cell lines (Figs. 7A–7D).

## Discussion

PROS1 is a cognate ligand of the TAM receptor-ligand complex. Increasing studies have demonstrated the vital roles of TAM receptor-ligand complex in inflammation, immunity, and cancer (Burstyn-Cohen & Maimon, 2019; Carrera Silva et al., 2013; Paolino et al., 2014; Paolino & Penninger, 2016; Rothlin et al., 2015), with the roles of TAM receptor *AXL* in PTC being proved by several independent research teams. For example, Avilla et al. (2011) and Collina et al. (2019) reported the TAM receptors *AXL* and growth arrest-specific six (*GAS6*) were overexpressed and activated in PTC samples. The association between *PROS1* and malignant tumors has also been defined by some researchers. Aberrantly high expression of *PROS1* displayed vital roles in promoting the development of glioblastoma, oral squamous cell carcinoma, and colorectal cancer (Abboud-Jarrous et al., 2017; Mat et al., 2016; Sierko et al., 2010). Moreover, two independent groups have preliminarily demonstrated the high expression of *PROS1* in PTC patients (Griffith et al., 2006; Zhang et al., 2019) applying bioinformatics methods. Similarly, this research also verified the high expression of *PROS1* in PTC samples compared with normal samples with several bioinformatics tools and *in vitro* experiments. Furthermore, based on the association between *PROS1* expression and clinicopathological features of PTC patients, the *PROS1* expression was found closely correlated with lymph nodes classification. Meanwhile, the cellular proliferation and migration ability were suppressed after *PROS1* knockdown, suggesting that *PROS1* promoted the proliferation and migration of PTC cells.

The popularity of *BRAF*^*V600E*^ mutation in TC has been shown in many studies. Studies have reported the association between *BRAF*^*V600E*^ mutation and aggressive cancer features, including lymph node metastases and recurrence (Cappola & Mandel, 2013; Ricarte-Filho et al., 2009). However, some studies failed to demonstrate the role of *BRAF*^*V600E*^ mutation in cancer pathogenesis (Chen et al., 2020; Yan et al., 2019a). Therefore, different investigations have displayed the contradictory roles of *BRAF*^*V600E*^ mutation in cancers. Even so, emerging studies have pointed that *BRAF*^*V600E*^ mutation is the most common genetic alteration affecting the PTC progression and prognosis, indicating that *BRAF*^*V600E*^ mutation could function as a major therapeutic target for PTC. Therefore, we want to evaluate the association between *PROS1* expression and *BRAF*^*V600E*^ allele in PTC patients. However, no significant relationship was observed between *PROS1* expression and *BRAF*^*V600E*^ mutation in PTC in our study.

AJCC system classifies DTC into four stages of mortality risk—I–IV, and it incorporates patient’s age at the diagnosis of cancer into the staging, using a cutoff age to separate the young and elder (Lamartina et al., 2018; Long et al., 2020). The cutoff age was 55 in the 8^th^ edition AJCC but 45 in the 6^th^ and 7^th^. The 8^th^ edition AJCC defines stage II as T1N1M0, T2N1M0 or T3NanyM0 of DTC in patients ≥ 55 years old, or any T/N with M1 in patients < 55 years old. Nowadays, stages III–IV and stages I–II have been defined as the advanced-stage and early-stage diseases, respectively (Perrier, Brierley & Tuttle, 2018). With the popularity of health checkups and the improvement of resolution of B-ultrasound in China, PTC can be detected at an early stage. In this study, only several patients at stage II were involved with no patients at stages III and IV. Therefore, more patients of high stages should be investigated in future studies.

The possible mechanisms of DEGs in cells were investigated based on GO and KEGG enrichment analyses (Liu et al., 2020; Niu et al., 2020; Yuan et al., 2020). GO enrichment analysis demonstrated that the DEGs between PTC and normal control samples might play roles in carcinogenesis through biological regulation, metabolic process, and binding-related mechanisms. Furthermore, KEGG pathway enrichment analysis illustrated that the DEGs between PTC and normal control samples were mainly enriched in several cancer-associated pathways, such as proteoglycans in cancer and focal adhesion pathways.

Though the present study verified that PROS1 was indeed overexpressed in PTC specimen and illustrated *PROS1* could act by promoting proliferation and migration in PTC cells, no detailed mechanisms through which *PROS1* could be enhancing the formation of PTC have been investigated here, and so no direct cause-effect link has been proven yet. In addition, the PTC patients involved in this study were mainly at stage I. Therefore, in the future studies, more patients of stages II–IV should be recruited to investigate the underlying roles of *PROS1* expression in PTC diagnosis and therapy.

## Conclusions

In this study, the upregulated PROS1 was identified in PTC tissues, by integrating bioinformatics methods and *in vitro* experiments, as the only immune-related hub gene to be deregulated in this type of cancer. Furthermore, PROS1 upregulation seems to be promoting PTC through increased proliferation and migration of the affected cells.

## Supplemental Information

10.7717/peerj.11813/supp-1 Supplemental Information 1 Sequences of sh*PROS1* and control shRNA.

10.7717/peerj.11813/supp-2 Supplemental Information 2 The clinicopathological information of patients with PTC.

10.7717/peerj.11813/supp-3 Supplemental Information 3 Normalization graphs of the 5 datasets. (A–E) The normalization graphs of GSE3678, GSE29265, GSE60542, GSE33630 and GSE3467, respectively.

10.7717/peerj.11813/supp-4 Supplemental Information 4 Analysis of *PROS1* expression of PTC and normal tissues in TCGA. The expression of *PROS1* is high in the PTC group compared with the normal control group. ***P < 0.001.

10.7717/peerj.11813/supp-5 Supplemental Information 5 *PROS1* was significantly knocked down by shRNAs. (A–B) RT-qPCR analysis results of *PROS1* expression in BCPAP and KTC-1 cells. (C–D) Western blot analysis results of *PROS1* expression in BCPAP and KTC-1 cells. **P < 0.01.

10.7717/peerj.11813/supp-6 Supplemental Information 6 QT-qPCR results of the PTC and normal group.

10.7717/peerj.11813/supp-7 Supplemental Information 7 The intersection of DEGs of the 5 datasets consisting of 347 DEGs.

10.7717/peerj.11813/supp-8 Supplemental Information 8 R code.

## Additional Information and Declarations

### Data Availability

The following information was supplied regarding data availability: The data are available at GEO: GSE3467, GSE3678, GSE29265, GSE33630, and GSE60542. The raw measurements and data are available in the Supplementary Files. The IHC pictures are available at figshare: Wang, Jing (2021): IHC.rar. figshare. Dataset. DOI 10.6084/m9.figshare.14627367.v1.
