# Supplementary material for: Aberrant expression of PROS1 correlates with human papillary thyroid cancer progression
Source: PeerJ. 2021 Aug 3;9:e11813. doi: 10.7717/peerj.11813 (PMC8344691; doi:10.7717/peerj.11813)
Supplement: Supplemental Information 1 [file peerj-09-11813-s001.docx]

Supplementary Table 1: Sequences of sh*PROS1* and control shRNA

| Gene name：PROS1 | Virus name：LV-PROS1 |
| --- | --- |
| ShPS1-a:5’-CCGGCCATGCAATGAAGATGGATATCTCGAGATATCCATCTTCATTGCATGGTTTTTG -3’ | |
| shPS1-b:5’-AATTCAAAAACCATGCAATGAAGATGGATATCTCGAGATATCCATCTTCATTGCATGG -3’ | |
| ShPS2-a:5’-CCGGCGTCGTGCAAATTCTTTACTTCTCGAGAAGTAAAGAATTTGCACGACGTTTTTG -3’  shPS2-b:5’-AATTCAAAAACGTCGTGCAAATTCTTTACTTCTCGAGAAGTAAAGAATTTGCACGACG -3’ | |
| CON313: TTCTCCGAACGTGTCACGT | |
