# Supplementary figures and images for: Aberrant expression of PROS1 correlates with human papillary thyroid cancer progression
Source: PeerJ. 2021 Aug 3;9:e11813. doi: 10.7717/peerj.11813 (PMC8344691; doi:10.7717/peerj.11813)

The expression of PROS1  
 $\text{Log}_2(\text{TPM}+1)$

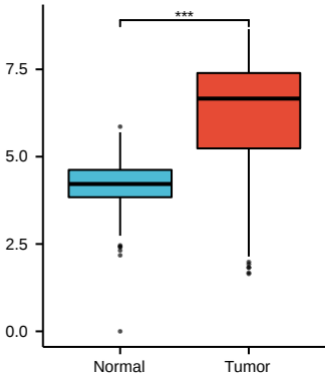

Supplement: Supplemental Information 4 — The expression of PROS1 is high in the PTC group compared with the normal control group. ***P < 0.001. [file peerj-09-11813-s004.pdf]
